# Assessment of BOLD and GenBank – Their accuracy and reliability for the identification of biological materials

**DOI:** 10.1371/journal.pone.0217084

**Published:** 2019-06-19

**Authors:** Kelly A. Meiklejohn, Natalie Damaso, James M. Robertson

**Affiliations:** 1 Counterterrorism and Forensic Science Research Unit, Visiting Scientist Program, Federal Bureau of Investigation Laboratory Division, Quantico, Virginia, United States of America; 2 Counterterrorism and Forensic Science Research Unit, Federal Bureau of Investigation Laboratory Division, Quantico, Virginia, United States of America; Chang Gung University, TAIWAN

## Abstract

Taxonomic identification of biological materials can be achieved through DNA barcoding, where an unknown “barcode” sequence is compared to a reference database. In many disciplines, obtaining accurate taxonomic identifications can be imperative (*e*.*g*., evolutionary biology, food regulatory compliance, forensics). The Barcode of Life DataSystems (BOLD) and GenBank are the main public repositories of DNA barcode sequences. In this study, an assessment of the accuracy and reliability of sequences in these databases was performed. To achieve this, 1) curated reference materials for plants, macro-fungi and insects were obtained from national collections, 2) relevant barcode sequences (*rbcL*, *matK*, *trnH-psbA*, *ITS* and *COI*) from these reference samples were generated and used for searching against both databases, and 3) optimal search parameters were determined that ensure the best match to the known species in either database. While GenBank outperformed BOLD for species-level identification of insect taxa (53% and 35%, respectively), both databases performed comparably for plants and macro-fungi (~81% and ~57%, respectively). Results illustrated that using a multi-locus barcode approach increased identification success. This study outlines the utility of the BLAST search tool in GenBank and the BOLD identification engine for taxonomic identifications and identifies some precautions needed when using public sequence repositories in applied scientific disciplines.

## Introduction

Over the last few decades, there has been a paradigm shift in the methods routinely used for the taxonomic identification of biological materials. Traditionally, identifications were achieved after rigorous examination of morphological characteristics and subsequent consultation with the appropriate authoritative taxonomic literature. However, in scenarios where the specimen is incomplete, traditional morphological methods can only generate reliable classifications at higher taxonomic levels. Considering molecular techniques are fast, more accessible and affordable, scientists globally are capitalizing on the discriminatory information contained in rapidly evolving regions of the genome to achieve species-level identifications.

Across the entire tree of life, a similar set of core gene regions, such as the nuclear internal transcribed spacers (*ITS*), ribosomal RNAs (*e*.*g*., 12S, 16S, and 18S), and protein-coding genes from the mitochondrial genome (*e*.*g*., cytochrome b [*cyb*], cytochrome oxidase subunit I and II [*COI* and *COII*] genes), have proven particularly useful for taxonomic discrimination. The fungal community has been using rDNA markers to identify unknown fungi since the 1990’s [1]. In 2003, Hebert and colleagues [2] coined DNA barcoding, in which they proposed that a 648 bp fragment of *COI* could permit species-level resolution among all animals. DNA barcoding has been broadly accepted as a reliable method of identification, and the barcoding concept has extended beyond animals; a 2-locus barcode of ribulose 1,5-biphosphate carboxylase (*rbcL*) and maturase K (*matK*) can resolve land plants [3] and a 645 bp region of *ITS* (spanning subunits 1 and 2) is used for fungi identifications [4]. One main advantage of DNA barcoding is that extensive public reference databases of barcode sequences already exist; an unknown sequence can easily be searched against a database to determine the closest species match (*e*.*g*., providing a measure of similarity).

The Barcode of Life DataSystems (BOLD) [5] and GenBank [6] are the two main public databases of DNA barcode data for animals, plants, and fungi. BOLD currently contains sequences for ~296,000 formally described species (~7 million specimens) (accessed 04/26/2019). For a sequence to obtain a ‘formal’ barcode status in BOLD, several elements must be provided [5]: species name, voucher data (storing institution and catalog information), collection record, identifier of the specimen, sequence of >500 bp, primer information, and the raw sequence data files. Once uploaded, BOLD administrators perform quality checks of data prior to making it public (*i*.*e*., confirmation the sequence is not that of a contaminant, is a true functional copy, and is of adequate quality) [5]. GenBank is much larger and contains >212 million sequences (accessed 04/26/2019). GenBank also performs basic quality checks on all new submissions, such as vector contamination, proper translation of coding regions, correct bibliographic citations and correct taxonomy. However, unlike BOLD, GenBank does not store sequence chromatograms, collection metadata or photographs [6]. BOLD is a curation tool that also stores sequences, while GenBank is just a sequence repository. Many sequences are duplicated between databases, as all BOLD sequences are automatically submitted to GenBank (denoted by the key term “BARCODE”) and BOLD periodically ‘mines’ barcode sequences from GenBank [7,8]. Ideally, all barcode sequences contained in either database should have been derived from a vouchered specimen, which was initially identified by a taxonomic expert. However, given the inherent nature of any public database, it is inevitable that some erroneous data will be present. The generation and submission of incorrect sequences likely occurs due to misidentification of the original material, poor isolation techniques (primarily for fungi), contamination of cultures, endoparasites in insects (*e*.*g*., *Wolbachia*) and plants (*e*.*g*., fungal endophytes), duplicate records due to instances of synonymy and PCR-based errors (*e*.*g*., chimeric sequences or the unintentional sequencing of pseudo-genes) [9–11]. Only a few studies have assessed the accuracy of sequence data contained in public databases, and these have largely been taxa specific [10,12–17]. For fungi, two independent studies completed over a decade ago estimated that up to 20% of the sequences contained in public databases are unreliable [10,13]. Additionally, these studies also highlighted that over 80% of the sequences in such databases lack reference to a vouchered specimen and are from environmental samples (*e*.*g*., uncultured from soil or root tips) [18].

Universal primer design and standard cycling conditions have meant that generating DNA barcode sequences is typically straight-forward across a broad range of taxa and sample types. Thus, DNA barcoding holds substantial potential for identifying potentially compromised unknown biological materials [19–21]. This study was aimed at performing an initial assessment of both the quality and reliability of data contained in BOLD and GenBank for obtaining taxonomic identifications of insect, macro-fungi, and plant taxa. To achieve this, curated reference material (n = 94) from the National Museum of Natural History (USNM; Washington, DC) and the U.S. Department of Agriculture Agricultural Research Service (USDA-ARS) U.S. National Fungus Collections (Beltsville, MD) were obtained and the appropriate barcode regions advocated for by the Consortium for the Barcode of Life (CBOL) (*i*.*e*., *COI*, *rbcL*, *matK*, *trnH-psbA*, *ITS*) amplified and sequenced. Using this data, we examined: 1) the accuracy and reliability of BOLD and GenBank as reference sequence databases for taxonomic identifications at the genus and species levels, and 2) the optimal set of algorithm parameters (*i*.*e*., BOLD identification engine and BLAST search methods) to use when searching BOLD and GenBank for reliable taxonomic identifications of unknown materials. Our aim was to test these databases as they are most commonly accessed and used in applied scientific disciplines. While there is overlap in the barcode sequences present in both databases, exclusion of such duplicate sequences (*i*.*e*., BOLD sequences from GenBank searches and vice versa) is not straight forward and would not be performed prior to routine searching. Thus, while our assessment compares both databases separately, they were not examined exclusively.

## Materials and methods

### Specimens

Curated reference material for insects (n = 17) and plants (n = 61) were obtained from the Entomology and Botany Departments of USNM. Macro-fungi (n = 16) were obtained from the USDA-ARS U.S. National Fungus Collections (Beltsville, MD). Taxa included in this study encompassed some that possess a forensic importance (*e*.*g*., plants and macro-fungi that produce poisonous toxins, along with insects commonly associated with decomposing corpses), but also those from a diverse range of orders and families. It should be noted that all taxa included in this study had pre-existing DNA barcode sequences in both BOLD and GenBank. Information on the specimens, including taxonomy and collection date, are given in S2–S4 Tables.

### DNA extraction

Prior to extraction, each tissue subsample was weighed using an AB304-S/FACT Analytical Balance (Mettler Toledo, Crescent, Singapore), with on average 0.9 (± 1.7), 18.3 (± 8.5) and 9.5 (± 5.9) mg of tissue used in insect, macro-fungi and plant extractions, respectively. Tissue was homogenized prior to extraction using a sterile Kimble Biomasher II closed system microtissue homogenizer 1.5 mL tube (Fisher Scientific, Hampton, NH, USA).

#### Insects

Total genomic DNA was isolated using the Qiagen DNeasy Blood and Tissue DNA Purification Kit (Qiagen, Hilden, Germany). The manufacturer’s protocols were followed for extraction with the following exceptions: 5 μL of Proteinase K (20 mg/mL; VWR International [E195], Radnor, PA, USA) was added to tissue homogenized in 75 μL of buffer ATL and incubated in a 56°C shaking water bath for 2 h; 50 μL of buffer AL and 50 μL of ethanol (96–100%; Sigma-Aldrich [E7023], St. Louis, MO, USA) were added following incubation at 56°C; the DNA was eluted in a single eluate of 50 μL of buffer AE, to maximize the final DNA concentration.

#### Macro-fungi and plants

A cetyltrimethylammonium bromide (CTAB) buffer was used for lysis and contained the following: 2% CTAB (VWR International [VWRV0833]), 100 mM Tris-HCl pH 8.0 (ThermoFisher [AM9855G], Waltham, MA, USA), 20 mM EDTA (Sigma-Aldrich [E7889]) and 1.4 M NaCl (ThermoFisher [AM9759]). Immediately prior to extraction, 0.04 g/mL polyvinylpyrrolidone (PVP; molecular weight of 360,000; Sigma-Aldrich [P5288]), 0.4% Proteinase K (20 mg/mL; VWR International [E195]), and 0.5% β-mercaptoethanol (Sigma-Aldrich [M3148]) was added to the CTAB lysis buffer, which was subsequently placed in a 56°C water bath for ~15 min to facilitate the dissolution of PVP. A total of 500 μL of the CTAB lysis buffer was added to the finely ground tissue and incubated in a 65°C shaking water bath for 1 hr. Following incubation, 500 μL of Phenol:Chloroform:Isoamylalcohol (25:24:1; Sigma-Aldrich [AM9732]) was added, mixed well and centrifuged for 10 min at 12,000 x *g*. The aqueous phase was transferred to a new sterile 1.5 mL tube containing 500 μL of 100% chloroform (Fisher Scientific [C606-1]) and mixed vigorously prior to centrifuging at 12,000 x *g* for 8 min. The aqueous phase was transferred to another new sterile 1.5 mL tube containing 900 μL of absolute ethanol (Sigma-Aldrich [E7023]) and placed in a -20°C freezer overnight (~18 hrs). After removal from the -20°C freezer, tubes were centrifuged for 5 min at 12,000 x *g* and all the liquid was carefully removed (as to not disturb the DNA pellet). The pellet was subsequently washed twice, once with 70% ethanol and once with absolute ethanol, as follows: 700 μL of ice cold ethanol (either 70% or absolute) was added, the tube was inverted once to mix, centrifuged for 1 min at 12,000 x *g* and all the liquid was carefully removed. The washed pellet was subsequently dried using a 55°C hot plate (~5 min) and re-suspended in 50 μL of TE Buffer (10mM Trizma HCl (Sigma-Aldrich [T3038]), 1mM EDTA (Sigma-Aldrich [E7889])). Extracts were purified with Agencourt AMPure XP beads (Beckman Coulter [A63880], Brea, CA, USA) per manufacturer’s recommendations for genomic DNA.

### Amplification, purification and quantification

All amplifications were performed on a GeneAmp PCR System 9700 Thermal Cycler (Applied Biosystems, Foster City, CA, USA). Using the manufacturer’s suggested reaction mix constituents, the Q5 Hot Start High-Fidelity DNA polymerase (New England BioLabs Inc [M0494S], Ipswich, MA, USA) was used for insect amplifications, and the KAPA3G Plant DNA polymerase (KAPA Biosystems [KK7251], Wilmington, MA, USA) for plant and macro-fungi amplifications. The barcode regions targeted for amplification were those adopted and advocated for use by the barcoding community and CBOL: 1) insects, *COI* [2]; 2) macro-fungi, *ITS* (subunits 1 and 2) [4]; and 3) plants, *rbcL* and *matK* [3]. Considering alternate loci have been identified by CBOL and are often used as supplemental markers for the identification of land plants, data for the intergenic spacer *trnH-psbA* and *ITS2* were also collected for plant taxa. S1 Table outlines the primer pairs and cycling conditions used in amplifications. Given that the taxa included in this study spanned numerous orders, successful amplification of regions such as *matK* and *trnH-psbA* was not possible for all taxa using a single primer pair; amplification of these regions were only achieved after screening with multiple primer pairs. PCR products were screened, purified and quantified as outlined in Meiklejohn *et al*. (2018) [21].

### Sequencing and data analysis

Sanger sequencing of plant and insect PCR amplicons was completed as outlined in Meiklejohn *et al*. (2018) [21] and macro-fungi *ITS* amplicons were processed following the same protocol used to sequence plants. After the removal of primer sequences and ambiguous bases using Sequencher v5.4.5 (Gene Codes, Ann Arbor, MI, USA), each reference sequence was queried against BOLD and GenBank using their built-in search tools (*i*.*e*., BOLD identification engine and GenBank’s BLAST). It is important to note that for macro-fungi, sequence data from *ITS* subunits 1 and 2 were searched both alone and in combination.

Unlike GenBank, the BOLD database is organized such that sequences for each taxonomic group are segregated (*i*.*e*., *COI* for animals, *rbcL* and *matK* for plants, and *ITS* for macro-fungi). Moreover, search algorithms behind BOLD and GenBank differ; BOLD searches the translated global protein sequence, while GenBank compares nucleotide query sequence to database sequences. Using the identification systems (IDS) search algorithm [5] in BOLD, each barcode sequence was searched against the appropriate collection of barcode sequences. In GenBank, a *MegaBlast* search, which is optimized for highly similar sequences and is the default nucleotide BLAST in the online interface, was used to search against all ‘other’ nucleotide sequences using the command-line interface (accessed July 2017- January 2018). This was facilitated through the use of a custom python script, which automated the submission of individual sequences for a *MegaBlast* search (provided upon request). The default settings for *MegaBlast* were employed as follows: 1) *max target sequence*, maximum number of aligned sequences to be written to the output file, 10; 2) *word size*, length of initial exact match, 28; and 3) *reward/penalty*, reward/penalty for a nucleotide match/mismatch, 1/-2 respectively [22]. The output from searches against both BOLD and GenBank were treated as follows: species were considered correctly identified (*i*.*e*., accurate) if a record with the same taxonomic name had the top match statistic (*i*.*e*., the lowest e-value, number of hits one can expect to see by chance when searching a database of a particular size; highest bit score, measures the sequence similarity independent of query sequence length and database size; and highest percent identity, percentage of similarity between two sequences). Query coverage (percent of query sequence that aligns to a sequence in GenBank) is another important metric that can be used to define good quality hits. However, as this metric is exclusive to GenBank, it was not examined in this study. Identifications were considered reliable if multiple independent records with the same top match statistics had the correct taxonomic name. In instances where multiple records with different taxonomic names had the same top match statistics (excluding known synonyms), the identification was treated as an ambiguous correct match.

In attempts to increase the stringency and reduce the ambiguity of identifications using both databases, modified searches were completed to identify the optimal set of algorithm parameters or sequence subsets. For each database, modifications were restricted to those available for use with either the online search tool (*i*.*e*., BOLD) or command line interface (*i*.*e*., GenBank). Assessing the impact of modifying the underlying algorithm, that could be possible with advanced computing and programmer resources (*e*.*g*., classification methods [k-mer, machine learning, phylogenetic hybrids] in conjunction with the databases), was not explored. For BOLD, the settings for the online IDS search algorithm cannot be configured. However, for *COI*, there are different subsets of sequences that can be searched: all barcode records on BOLD, only species-level barcode records (default), only public barcode records, only full-length barcodes (>640 bp). Thus, all *COI* sequences generated in this study were searched against these various sequence subsets. For GenBank, *blastn* searches against the ‘other’ nucleotide sequences were completed using the command-line interface, with modifications to the *word size* and *reward/penalty* (defaults of 11 and 2/-3, respectively). These two parameters were chosen for modification as they are linked to the specificity of sequence matches: 1) a large *word size* (*e*.*g*., 28, the *MegaBlast* default) is optimized for intra-species comparison (~99% conserved), while a shorter *word size* (*e*.*g*., 11, the *blastn* default) is better suited for inter-species comparisons (~95% conserved); 2) a *reward/penalty* ratio of 1/-2 is optimized for inter-species comparisons, whereas a ratio of 1/-3 is better suited for intra-species comparisons [22]. In this study, the following parameters were independently modified for *blastn* searches (all other parameters remained at the default settings): 1) *word size* of 17, 2) *reward/penalty* of 1/-2, and 3) *reward/penalty* of 1/-3. Two-sample t-tests and one-way ANOVAs were used to determine if there was a statistically significant difference between the two public databases and between the different search parameters tested for each taxonomic group.

## Results and discussion

### Insects

Amplification of the full-length *COI* barcode region was initially attempted for all 17 insect taxa representing 12 orders (using the primer pairs outlined in S1 Table). Amplicons of the expected size were only obtained for 12 taxa (average length, 614 ± 34 bp). This reduced amplification success can be attributed to the material available for extraction (*e*.*g*., only single legs, no flight muscle), such that the DNA extracted likely was highly degraded restricting the amplification of the full *COI* barcode region. For the five taxa that failed to amplify the full region, amplifications of smaller barcode regions were sequentially attempted (*i*.*e*., ~250–400 bp; S2 Table) and were successful (average length, 340 ± 65 bp).

**Fig 1:**
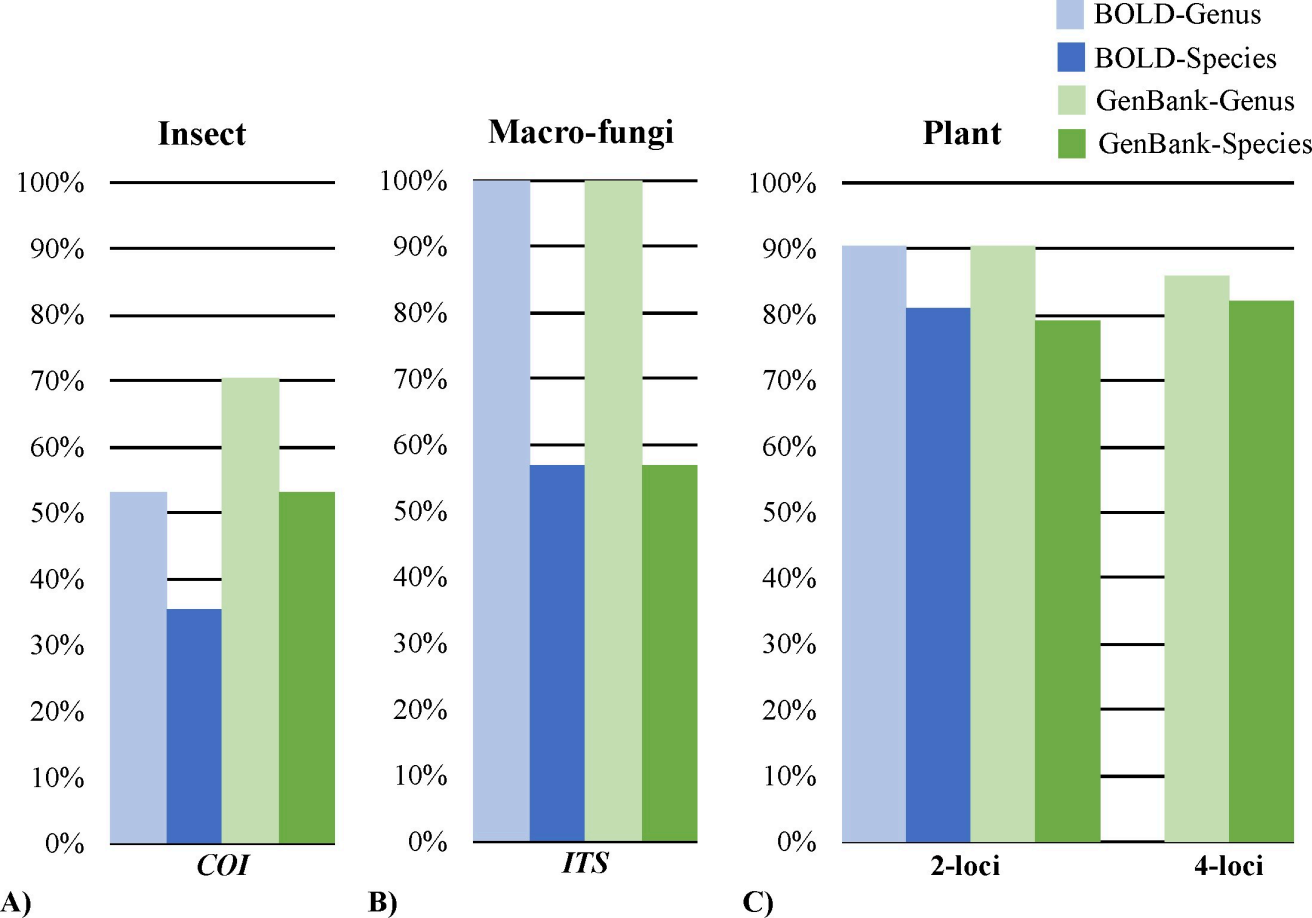
**Overall classification accuracies from BOLD and GenBank for: A) *COI* insect sequences (n = 17), B) *ITS* macro-fungi sequences (n = 14), and C) plant taxa using either a 2-locus (*rbcL* and *matK*; n = 53) and 4-locus approach (*rbcL*, *matK*, *trnH-psbA* and *ITS2*; n = 28)**. The identification success for genus is denoted by the light color and species by the dark color. Blue bars correspond to results from searches against BOLD and green against GenBank.

**Fig 2:**
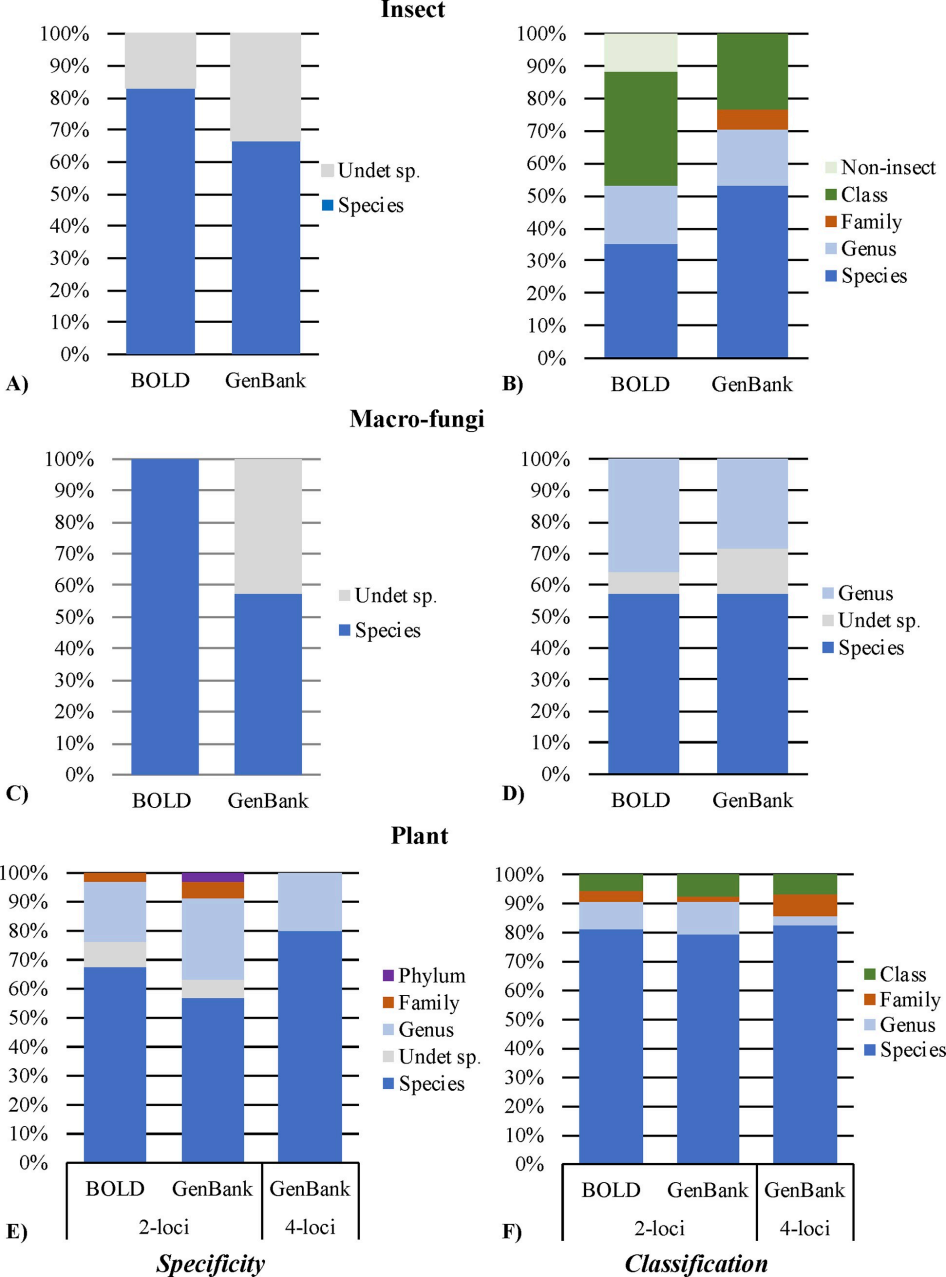
**Classification using BOLD and GenBank for: A-B) *COI* insect sequences (n = 17), C-D) *ITS* macro-fungi sequences (n = 14), and E-F) plant taxa using either a 2-locus approach (*rbcL* and *matK*; n = 53) or 4-locus approach (*rbcL*, *matK*, *trnH-psbA*, and *ITS2*; n = 28)**. A,C,E) Assessment of the specificity of the top match(es) in both databases: reliable match, where all records with the same top match statistics matched the expected taxa (dark blue), or ambiguous match, where records with the same top statistic match represent more than one species (other colors; *e*.*g*., gray = undetermined species, light blue = congeneric species, *etc*). B,D,F) Taxonomic level classification. Taxa were correctly identified to the species-level (dark blue) or higher taxonomic level (other colors; *e*.*g*., light blue = genus, green = class, *etc*).

Of the 17 sequences queried against both databases, GenBank outperformed BOLD for both genus and species level identifications, although this failed to meet statistical significance (*p*>0.30) (Fig 1A). For both databases, <70% of taxa were correctly assigned at both the genus and species level (Fig 1A). This result was lower than previously reported for flies (Diptera [15,20], beetles (Coleoptera [23]), butterflies and moths (Lepidoptera [24]), when searching against either or both of these databases. Six taxa were classified as being an ambiguous match (*i*.*e*., multiple species, including the expected, with the same top match statistics) when searching against both databases (Fig 2A). For taxa that were misidentified at the species level in BOLD and GenBank (n = 8), correct classification at higher taxonomic levels (*i*.*e*., genus, family, order, class) was still achieved in the majority of cases (Fig 2B). The BOLD percent of similarity statistic was identified as an accurate indicator of an incorrect match (*p* = 2.02x10^-4^), while the comparable GenBank similarity statistic (*i*.*e*., percent identity) was not. For example, in instances where taxa were misidentified, the BOLD percent similarity statistic was 60 ± 15%, whereas the percent identity from GenBank was 98 ± 2%. It should be noted that although there are differences in how BOLD and GenBank search their respective databases (*i*.*e*., translated global protein alignments and pairwise nucleotide alignments for BOLD and GenBank, respectively), both similarity statistics define the underlying number of nucleotide differences between the query and reference.

Low species level identification success was unexpected given that taxa were chosen in part based on their inclusion in BOLD and GenBank, but also given that insects have been well characterized using DNA barcoding (~76% and 75% of all *COI* animal sequences in BOLD and GenBank, respectively are from insects [accessed 11/26/2018]). Species-level misidentifications could be attributed to the inclusion of misidentified specimens in public databases, given that morphological identifications between closely related species are challenging in many orders. For example, *Chrysomya saffranea* and *Chrysomya megacephala* (Diptera: Calliphoridae) [25], *Eurygaster maura* and *Eurygaster testudinaria* (Hemiptera: Scutelleridae) [26], *Appasus japonicus and Appasus major* (Heteroptera: Belostomatidae) [27] are commonly misidentified morphologically. It is also possible that, given approximately 30% of the *COI* sequences obtained in this study were less than 430 bp, the discriminatory power needed to facilitate species-level identifications may have been diminished (reduced query length often negatively affects the ability to get a good match; S2 Table). Nevertheless, Grywacz and colleagues [20] examined the impact of barcode sequence length on identification success in Diptera. In their study, they applied a best match criteria with SpeciesIdentifier v1.8 using sequences in BOLD and GenBank relating to Dipteran Family (Fannidae), and obtained similar results when using fragments of 650 bp and 130 bp (96% and 94%, respectively). In this study, misidentifications were not observed exclusively for taxa in which a mid-length *COI* barcode sequence was used to search against public databases. Species-level misidentification rates of approximately 42% and 60% were observed when using full- and mid-length sequences, respectively, for searching against either public database (S2 Table).

### Macro-fungi

Using a range of primer pairs, the amplification and sequencing of *ITS1* and *ITS2* was successful; average separate lengths of 241 ± 73 bp and 295 ± 52 bp were obtained respectively, with a combined average length of 536 ± 93 bp (S3 Table). As complete high-quality sequence data for both *ITS1* and *ITS2* were not obtained for *Amanita ocreata* and *Conocybe filaris* (S3 Table), these taxa were excluded from analyses. Searches against BOLD and GenBank were completed for *ITS1* and *ITS2* both alone and in combination. Similar to results observed by Porras-Alfaro and colleagues [28], no significant difference (*p*>0.59) in the discrimination power between *ITS1* and *ITS2* was observed (S3 Table).

When searching both databases with the combined *ITS1* and *ITS2* sequence, all taxa were assigned to the correct genus and the average similarity percentage of top matches was 98 ± 3%. Correct species level identifications were only achieved for 57% of taxa, when searching against either BOLD or GenBank (Fig 1B). For correctly identified taxa that had multiple records with the same top match statistics (n = 3 and 7 for BOLD and GenBank, respectively), ambiguous matches were only observed when searching against GenBank (albeit only 43% of the time) (Fig 2C). In these instances, the additional records were from uncultured or undetermined species. For the eight taxa that were misidentified at the species level, these represented five of nine genera sampled in this study. These misidentifications were not restricted to a single database; four were misidentified in both databases, two from BOLD and two from GenBank (S3 Table). In these instances, the record(s) with the top match statistics were either a congeneric species (*i*.*e*., different species from the same genus) or an undetermined species of the correct genus (*e*.*g*., *Amanita* sp.) (Fig 2D).

It has been documented that the ~97,000 currently described fungi taxa likely only represent ~2% of all existing species [29]. Given this, it is not surprising that public databases include a high proportion of records that are undetermined or uncultured, limiting their utility for species-level identifications [18,30–31]; species names are not given for ~11% and 48% of *ITS* sequences found in BOLD and GenBank, respectively (accessed 11/26/2018). Aside from the difficulties with documenting the breadth of fungi biodiversity, accurate identification of adequately described species is challenging for non-experts, as only subtle morphological variations may separate species and the *ITS* region is not equally variable among all groups of fungi [13,32–34]. Additionally, the generation of clean, reliable sequence data from fungal cultures can be difficult, as cross-contamination from symbiotic organisms is possible [10].

### Plants

Given the more conserved nature of the *rbcL* locus [35], amplification and sequencing were successful for all but one species sampled in this study (S4 Table). In contrast, the generation of barcode sequences for *matK*, *trnH-psbA*, and *ITS2* was not as seamless, even after screening with multiple primer pairs (S1 Table); high-quality sequences were only generated for 85%, 78%, and 73% of taxa, respectively (S4 Table). Considering CBOL currently only advocates for the use of a 2-locus barcode for species-level identifications in land plants [3] (despite listing supplemental barcode loci), BOLD only currently contains *rbcL* and *matK* sequences. In this study, reference barcode sequences from *rbcL* and *matK* were searched against both BOLD and GenBank, but *trnH-psbA* and *ITS2* sequences were only able to be searched against GenBank.

No significant difference (*p*>0.48) was observed in the discrimination power at the genus and species levels between the four loci (average accuracy, 79 ± 6% and 63 ± 5%, respectively). Additionally, for *rbcL* and *matK*, no significant difference (*p*>0.70) in accuracy at either taxonomic level was observed between BOLD and GenBank. Despite *ITS2* having the lowest discrimination power in this study, previous studies have documented successful identifications using only *ITS2* for medicinal plants [36], daisies (Asteraceae) [37] and citrus (Rutaceae) [38]. Furthermore, for species that lack chloroplasts (*e*.*g*., parasitic plants), *ITS2* can be an informative marker to permit species identifications.

When examining the accuracy of identifications using the CBOL recommended 2-locus barcode (n = 53), there was no significant difference between BOLD and GenBank (*p*>0.82), with correct identifications to genus and species obtained for approximately 91 ± 0% and 80 ± 1%, respectively (Fig 1C). Park and colleagues (2017) [39] also reported similar success when using the 2-locus barcode for the species-level identification of thirty land plants (84%). For taxa that were correctly identified but had multiple records with the same top match statistics (n = 34–35), 68% and 57% of these matches were considered unambiguous from BOLD and GenBank, respectively (Fig 2E). In instances where taxa were incorrectly identified at the species level, assignment at higher taxonomic levels was still possible using either database (Fig 2F).

Sequences for all 4 barcode loci were obtained for 28 taxa, with the sequences subsequently searched against GenBank. Greater resolution was achieved using the 4-locus barcode, with correct identification to genus and species for 86% and 82%, respectively. For correctly identified taxa that had multiple records with the same top match statistics (n = 15), 80% of these matches were considered unambiguous (*i*.*e*., all multiple records corresponded to the known species; Fig 2F). To date, there has not been any other studies that have examined the utility of the combined 4-locus barcode for species identification when searching against GenBank. This study demonstrates that adopting the 4-locus barcode for land plants not only provides better discrimination, but also decreases the ambiguity of identifications (when compared to the 2-locus approach) (Fig 2E).

### Database search parameters

The experimental design of this study enabled the assignment of correct matches as either unambiguous (*i*.*e*., all top matches were to the expected species of the vouchered specimens) or ambiguous (*i*.*e*., multiple species, including the expected, with the same top match statistics of the top ten in the output). When searching against both BOLD and GenBank, 22 ± 12% of all correct matches were classified as ambiguous. In a scenario where no prior taxonomic information about a sample is known, it would be difficult to discern the correct species if multiple records representing numerous species had the same top match statistics. To address this, we assessed whether applying more stringent search parameters would reduce the number of ambiguous correct matches from both BOLD and GenBank.

Modified searches against BOLD were only possible for *COI*, in which same algorithm is used to query four different sequence subsets. When comparing the output from the four subsets, no significant differences were observed with respect to number of correct species level classifications (*p* = 0.12) and ambiguity (*p* = 0.22). However, there was a significant difference in correct genus level classifications (*p* = 0.002) and similarity percentages (*p* = 4.99x10^-3^). For sequences that returned <96% similarity against the “all” sequence subset, a “no match” was obtained from the other sequence subsets (*i*.*e*., full, public and species). Therefore, it is recommended to search an unknown against the “all” sequence subset (rather than the default ‘species’ sequence subset), and use the percent similarity to determine the confidence of a match. A total of four different searches were completed against GenBank for all sequences; the default *MegaBlast* along with three modified *blastn* searches. When comparing the output from these searches in GenBank for all three taxonomic groups, no significant differences were observed with respect to the number of correct genus and species level classifications, the number of correct ambiguous matches, or the top match statistics (*i*.*e*., percent similarity, score).

In this study, the modified searches against public databases did not effectively decrease the number of ambiguous correct matches. It should be noted that this study solely looked at straight-forward modifications to the existing search parameters provided by BOLD and GenBank, to reflect how the majority of users would interact with these databases. Further advanced classification tools can be utilized in conjunction with BOLD and GenBank to increase taxonomic confidence (*e*.*g*., machine learning, RDP classifier, Pro-Tax) [40–41]. Additionally, others have developed tools to locally merge sequences from GenBank and BOLD to maximize taxonomic coverage and reliability [42]. Although modifications were not exhaustively tested in this study, a more straight-forward solution to address ambiguous correct matches could be to expand the number of sequences in public databases [8,31]. Efforts should be focused not only on obtaining full-length sequences, but also on obtaining sequences to fill intra- and inter-specific gaps. The importance of having a complete reference database for searching, where the intra-specific and inter-specific variation is adequately characterized, was demonstrated by Wilkinson and colleagues [43]. In their study, when *rbcL* and *matK* sequence databases were incomplete, erroneous species-level identifications were observed in up to 40% of cases. While erroneous sequences cannot be easily removed from GenBank, efforts should be directed to expand the taxonomic coverage in curated databases by generating barcode sequences from vouchered material [14].

## Conclusions

This study demonstrated that the uncurated GenBank did not underperform, with respect to the number of correct genus and species level identifications, when compared to the well curated BOLD. Ambiguous correct matches were observed when searching against either database. In scenarios where the sequence represents a complete unknown, accurately interpreting and reporting on an ambiguous correct match poses a challenge. Modifying the search algorithms (GenBank) or the sequences used in searches (BOLD) did not significantly reduce correct match ambiguity. Rather, using a multi-barcode approach in plants and macro-fungi reduced ambiguity (*e*.*g*., 4-loci as opposed to 2-loci in plants, and 2-loci for macro-fungi) and enabled reliable species-level identifications; using multiple loci for insects is expected to have similar favorable results. Another solution to address concerns with incorrect species identifications would be to report the taxonomy at a higher level; accurate identifications to the genus and family levels were obtained using either database in this study. This study highlights some of the precautions that should be taken when using public sequence databases to identify unknown non-human biological materials, especially prudent in applied scientific disciplines.

## Supporting information

S1 File BOLD and GenBank output for samples used in statistical analyses (insects, n = 17; macro-fungi, n = 14; plants 2-loci, n = 53; plants 4-loci, n = 28). (doi: 10.6084/m9.figshare.8183063).
(XLSX)

S1 Table Primers and thermal cycling conditions used to amplify each barcoding region for insects, macro-fungi, and plants. (doi: 10.6084/m9.figshare.8171147).
(PDF)

S2 Table Specimen information and barcode results for insects. (doi: 10.6084/m9.figshare.8182631).
(PDF)

S3 Table Specimen information and barcode results for macro-fungi. (doi: 10.6084/m9.figshare.8182700).
(PDF)

S4 Table Specimen information and barcode results for plants. (doi: 10.6084/m9.figshare.8182703).
(PDF)
