# Supplementary material for: Assessment of BOLD and GenBank – Their accuracy and reliability for the identification of biological materials
Source: PLoS One. 2019 Jun 19;14(6):e0217084. doi: 10.1371/journal.pone.0217084 (PMC6584008; doi:10.1371/journal.pone.0217084)
Supplement: S1 Table — (doi: 10.6084/m9.figshare.8171147). (PDF) [file pone.0217084.s002.pdf]

S1 Table. Primers and thermal cycling conditions used to amplify each barcoding region for insects, macro-fungi, and plants.

Group	Barcode Region	Primers	Cycling conditions
**Insects**	*COI*	LCO1490-L/HCO2198-L *(1)*	1X 95°C for 2 min<br>40X 94°C for 30 sec, 52°C for 30 sec, 72°C for 2 min<br>1X 72°C for 5 min
		LCO1490/HCO2198 *(2)*	1X 95°C for 2 min<br>43X 94°C for 30 sec, 56°C for 30 sec, 72°C for 1 min<br>1X 72°C for 5 min
		LepF1/LepR1 *(3)*	1X 95°C for 2 min<br>5X 94°C for 40 sec, 45°C for 40 sec, 72°C for 70 sec<br>35X 94°C for 40 sec, 51°C for 40 sec, 72°C for 70 sec<br>1X 72°C for 5 min
		LepF1/MLepR1 *(4)* -35 cycles<br>MLepF1/LepR1 *(4)* -38 cycles	1X 95°C for 2 min<br>5X 94°C for 30 sec, 45°C for 40 sec, 72°C for 1 min<br>35-38X 94°C for 30 sec, 51°C for 40 sec, 72°C for 1 min<br>1X 72°C for 5 min
**Macro-fungi**	*ITS1*<br>*ITS2*	ITS5/ITS2 *(5)*<br>ITS3/ITS4 *(5)*	1X 94°C for 3 min<br>25-40X 94°C for 30 sec, 55°C for 30 sec, 72°C for 45 sec<br>1X 72°C for 10 min
**Plants**	*rbcL*	rbcLaF *(6)* /rbcLaR *(7)* -40 cycles<br>rbcLaF *(6)* /rbcLajf634R *(8)* -35 cycles	1X 95°C for 4 min<br>35-40X 94°C for 30 sec, 55°C for 30 sec, 72°C for 1 min<br>1X 72°C for 10 min
		rbcLa-1/rbcLa-B *(9)*	1X 95°C for 3 min<br>10X 95°C for 30 sec, 48°C for 30 sec, 72°C for 30 sec<br>25X 88°C for 30 sec, 52°C for 30 sec, 72°C for 30 sec<br>1X 60°C for 10 min
	matK	matK-KIM1R-F/matK-KIM3F-R *(10,11)*<br>matK-xf *(12)* /matKMALPR1 *(13)*	1X 94°C for 1 min<br>35X 94°C for 30 sec, 57°C for 20 sec, 72°C for 10 sec<br>1X 72°C for 5 min
		matK390F/matK1326R *(14)*	1X 98°C for 2 min<br>35X 98°C for 10 sec, 54°C for 30 sec, 72°C for 40 sec<br>1X 72°C for 10 min
		Gym_F1A/Gym_R1A *(15)*	1X 94°C for 3 min<br>35X 94°C for 30 sec, 52°C for 30 sec, 72°C for 45 sec<br>1X 72°C for 10 min
		moss404F/moss1336R *(16)*<br>polypodF1/polypodR1 *(17)*<br>polypodF2/polypodR1*(17)*	1X 98°C for 2 min<br>35X 98°C for 10 sec, 50°C for 30 sec, 72°C for 40 sec<br>1X 72°C for 10 min
	trnH-psbA	psbAF *(18)* /trnH2 *(19)*<br>psbA/trnH *(20)*	1X 98°C for 45 sec<br>35X 98°C for 10 sec, 64°C for 30 sec, 72°C for 40 sec<br>1X 72°C for 10 min
	ITS2	ITS2F *(21)* /ITS4 *(5)*	1X 94°C for 5 min<br>35X 94°C for 30 sec, 56°C for 30 sec, 72°C for 45 sec<br>1X 72°C for 10 min

References

1 Huemer P, Mutanen M, Sefc KM, & Hebert PD. Testing DNA barcode performance in 1000 species of European Lepidoptera: large geographic distances have small genetic impacts. PLoS One. 2014;9: e115774.

2 Folmer O, Black M, Hoeh W, Lutz R, & Vrijenhoek R. DNA primers for amplification of mitochondrial cytochrome c oxidase subunit I from diverse metazoan invertebrates. Mol. Marine Biol. Biotechnol. 1994;3: 294-299.

3 Hebert PD, Penton EH, Burns JM, Janzen DH, & Hallwachs W. Ten species in one: DNA barcoding reveals cryptic species in the neotropical skipper butterfly Astraptes fulgerator. PNAS. 2004;101: 14812-14817

4 Hajibabaei M, Janzen DH, Burns JM, Hallwachs W, & Hebert PD. DNA barcodes distinguish species of tropical Lepidoptera. PNAS. 2006;103: 968-971.

5 White TJ, Bruns T, Lee S, & Taylor J. Amplification and direct sequencing of fungal ribosomal RNA genes for phylogenetics. In: Innis MA, Gelfand DH, Sninsky JJ, White TJ., editors. PCR Protocols: A Guide to Methods and Applications. Cambridge: Academic Press; 1990. pp 315-322.

6 Levin RA, Wagner WL, Hoch PC, Nepokroeff M, Pires JC, Zimmer EA, et al. Family-Level Relationships of Onagraceae Based on Chloroplast rbcL and ndhF Data. Am. J. Bot., 2003;90:107-115.

7 Kress WJ, Erickson DL, Jones FA, Swenson NG, Perez R, Sanjur O, et al. Plant DNA barcodes and a community phylogeny of a tropical forest dynamics plot in Panama. PNAS. 2009;106: 18621-18626.

8 Fazekas AJ, Burgess KS, Kesanakurti PR, Graham SW, Newmaster SG, Husband BC, et al. Multiple multilocus DNA barcodes from the plastid genome discriminate plant species equally well. PLoS One. 2008;3: e2802.

9 Little DP. A DNA mini-barcode for land plants. Mol. Ecol. Res. 2014;14: 437-446.

10 Fazekas AJ, Kuzmina ML, Newmaster SG, & Hollingsworth, PM. Methods in Molecular Biology. In: Erickson D, Kress W, editors. DNA Barcodes Methods and Protocols. New York: Humana Press; 2012. pp. 223-252.

11 Costion C, Ford A, Cross H, Crayn D, Harrington M, Lowe A. Plant DNA barcodes can accurately estimate species richness in poorly known floras. PLoS One. 2011;6: e26841.

12 Ford CS, Ayres KL, Toomey N, Haider N, Stahl JVA, Kelly LJ, et al. Selection of candidate coding DNA barcoding regions for use on land plants. Bot. J. Linn. Soc. 2009;159: 1-11.

13 Dunning LT, & Savolainen V. Broad-scale amplification of matK for DNA barcoding plants, a technical note. Bot. J. Linn. Soc. 2010;164: 1-9.

14 Cuénoud P, Savolainen V, Chatrou LW, Powell M, Grayer RJ, Chase MW. Molecular phylogenetics of Caryophyllales based on nuclear 18S rDNA and plastid rbcL, atpB, and matK DNA sequences. Am. J. Bot. 2002;89: 132-144.

15 Li Y, Gao LM, Poudel RC, Li DZ, & Forrest A. High universality of matK primers for barcoding gymnosperms. J. Syst. Evol. 2011;49: 169-175.

16 Forrest A. DNA Barcoding of Plants: matK primers for mosses. (Royal Botanic Garden Edinburgh, Edinburgh, U.K., 2012).

17 Forrest, A. DNA Barcoding of Plants: matK primers for ferns and allies. (Royal Botanic Garden Edinburgh, Edinburgh, U.K., 2012).

18 Sang T, Crawford DJ, & Stuessy TF. Chloroplast DNA phylogeny, reticulate evolution, and biogeography of Paeonia (Paeoniaceae). Am. J. Bot. 1997;84: 1120-1136.

19 Tate JA, & Simpson BB. Paraphyly of Tarasa (Malvaceae) and diverse origins of the polyploid species. Syst. Bot. 2003;28: 723-737.

20 Hamilton M. Four primer pairs for the amplification of chloroplast intergenic regions with intraspecific variation. Mol. Ecol. 1999;8: 521-523.

21 Li X, Yang Y, Henry RJ, Rossetto M, Wang Y, Chen S. Plant DNA barcoding: from gene to genome. Biol. Rev. 2015;90: 157-166.
