# Supplementary material for: Assessment of BOLD and GenBank – Their accuracy and reliability for the identification of biological materials
Source: PLoS One. 2019 Jun 19;14(6):e0217084. doi: 10.1371/journal.pone.0217084 (PMC6584008; doi:10.1371/journal.pone.0217084)
Supplement: S2 Table — (doi: 10.6084/m9.figshare.8182631). (PDF) [file pone.0217084.s003.pdf]

S2 Table: Specimen information and barcode results for insects.

Order	Family	Genus	Species	Year	*COI*				
					Sequencing Primer	Query Length	GenBank Accession	BOLD	GenBank
Coleoptera	Scarabaeidae	*Phanaeus*	*vindex*	1996	LCO1490-L/HCO2198-L	581	MK905407	✓	✓
Dermaptera	Spongiphoridae	*Forficula*	*auricularia*	1993	LepR1	429	MK905402	X	✓✓+
Diptera	Calliphoridae	*Calliphora*	*vicina*	2007	LCO1490-L/HCO2198-L	553	MK905397	✓✓	✓✓
		Chrysomya	*rufifacies*	1989	LCO1490-L/HCO2198-L	592	MK905396	✓✓	✓✓
	Culicidae	*Aedes*	*aegypti*	1971	LepF1/MLepR1; MLepF1/LepR1	658	MK905393	✓✓+	✓✓
	Glossinidae	*Glossina*	*palpalis*	2000	LCO1490-L/HCO2198-L	611	MK905403	✓	✓
	Muscidae	*Musca*	*domestica*	1991	LCO1490/HCO2198	645	MK905404	X	X
Ephemeroptera	Ephemeridae	*Hexagenia*	*limbata*	-	LepF1/MLepR1	254	MK905400	X	X
Hymenoptera	Vespidae	*Vespula*	*squamosa*	2007	LCO1490-L/HCO2198-L	560	MK905409	✓✓	✓✓
Lepidoptera	Nymphalidae	*Danaus*	*plexippus*	1991	LCO1490-L/HCO2198-L	623	MK905401	✓✓	✓✓+
	Sarturniidae	*Callosamia*	*promethea*	1985	LepF1/MLepR1	354	MK905395	✓✓	✓✓
Mecoptera	Meropeidae	*Merope*	*tuber*	2008	LCO1490-L/HCO2198-L; LepF1/MLepR1	655	MK905405	X	✓✓
Neuroptera	Ascalaphidae	*Ululodes*	*quadripunctatus*	1984	LCO1490-L/HCO2198-L	635	MK905408	X	✓✓
Odonata	Gomphidae	*Gomphus*	*exilis*	2016	LCO1490/HCO2198	612	MK905399	X	X
Orthoptera	Gryllidae	*Gryllus*	*assimilis*	1985	LepF1/MLepR1	278	MK905398	✓	✓
Phthiraptera	Pediculidae	*Pediculus*	*humanus capitis*	1955	LepR1	384	MK905406	X	X
Siphonaptera	Pulicidae	*Ctenocephalides*	*felis*	1968	LCO1490-L/HCO2198-L	643	MK905394	X	X

X= misidentified at both the genus and species level; ✓= accurate genus level identification; ✓✓= unambiguous genus and species level identification; ✓✓+= ambiguous species level identification (*i.e.,* where records with the same top statistic match represent more than one species
