# Supplementary material for: Assessment of BOLD and GenBank – Their accuracy and reliability for the identification of biological materials
Source: PLoS One. 2019 Jun 19;14(6):e0217084. doi: 10.1371/journal.pone.0217084 (PMC6584008; doi:10.1371/journal.pone.0217084)
Supplement: S3 Table — (doi: 10.6084/m9.figshare.8182700). (PDF) [file pone.0217084.s004.pdf]

S3 Table: Specimen information and barcode results for macro-fungi.

Family	*Genus*	Species	Year	*ITS1*					*ITS2*					*ITS1 & ITS2*		
				Sequencing Primer	Query Length	GenBank Accession	BOLD	GenBank	Sequencing Primer	Query Length	GenBank Accession	BOLD	GenBank	Query Length	BOLD	GenBank
Amanitaceae	*Amanita*	*bisporigera*	1973	ITS5/ITS2	235	MK895671	✓	✓	ITS3/ITS4	149	MK895685	✓	✓	384	✓	✓
		gemmata	1967	ITS2	124	MK895672	✓	✓✓+	ITS4	340	MK895686	✓	✓✓	464	✓	✓✓
		ocreata	1982	N/A	N/A	N/A	N/A	N/A	ITS4	337	MK900748	✓✓	✓✓	N/A	N/A	N/A
		pantherina	1973	ITS5/ITS2	241	MK895673	✓✓	✓✓+	ITS4	241	MK895687	✓✓	✓✓+	482	✓✓	✓✓+
		phalloides	1975	ITS5/ITS2	237	MK895674	✓✓	✓✓+	ITS4	347	MK895688	✓✓+	✓✓+	584	✓✓	✓✓+
		verna	1969	ITS5/ITS2	219	MK895675	✓	✓	ITS4	321	MK895689	✓	✓	540	✓	✓
		virosa	1979	ITS2	112	MK895676	✓	✓✓+	ITS4	286	MK895690	✓	✓	398	✓	✓
Bolbitiaceae	*Conocybe*	*filaris*	1973	ITS5/ITS2	283	MK900745	✓✓	✓	N/A	N/A	N/A	N/A	N/A	N/A	N/A	N/A
	Panaeolina	*foenisecii*	1981	ITS5/ITS2	236	MK895682	✓✓	✓✓+	ITS3	235	MK895696	✓✓	✓	471	✓✓	✓
Clavicipitaceae	*Claviceps*	*purpurea*	1945	ITS5/ITS2	189	MK895677	✓✓	✓✓	ITS3/ITS4	290	MK895691	✓✓	✓✓	479	✓✓	✓✓
Discinaceae	*Gyromitra*	*esculenta*	1963	ITS5/ITS2	429	MK895679	✓	✓✓	ITS3/ITS4	318	MK895693	✓	✓✓	747	✓	✓✓
Hymenogastraceae	*Psilocybe*	*mexicana*	1958	ITS5/ITS2	242	MK895684	✓✓	✓✓	ITS4	319	MK895698	✓✓	✓✓	561	✓✓	✓✓
Inocybaceae	*Inocybe*	*rimosa*	1949	ITS5/ITS2	274	MK895680	✓✓	✓	ITS3/ITS4	324	MK895694	✓✓	✓	598	✓✓	✓
		geophylla	1982	ITS5/ITS2	306	MK895681	✓✓+	✓✓+	ITS3/ITS4	318	MK895695	✓✓	✓✓+	624	✓✓	✓✓+
Strophariceae	*Stropharia*	*cubensis*	1977	ITS5/ITS2	266	MK895683	✓✓+	✓✓+	ITS3/ITS4	333	MK895697	✓✓	✓✓	599	✓✓	✓✓
Tricholomataceae	*Clitocybe*	*rivulosa*	1963	ITS5/ITS2	261	MK895678	✓	✓	ITS3/ITS4	308	MK895692	✓	✓	569	✓	✓

N/A=specimen did not amplify with any primer pair or had low quality sequence data, thus no downstream analyses were possible; ✓= accurate genus level identification; ✓✓= unambiguous genus and species level identification; ✓✓+= ambiguous species level identification (*i.e.,* where records with the same top statistic match represent more than one species).
