# Supplementary material for: Assessment of BOLD and GenBank – Their accuracy and reliability for the identification of biological materials
Source: PLoS One. 2019 Jun 19;14(6):e0217084. doi: 10.1371/journal.pone.0217084 (PMC6584008; doi:10.1371/journal.pone.0217084)
Supplement: S4 Table — (doi: 10.6084/m9.figshare.8182703). (PDF) [file pone.0217084.s005.pdf]

S4 Table: Specimen information and barcode results for plants.

Group	Order	Family	Genus	Species	Year	# loci used	*rbcL* Sequencing Primer	*rbcL* GenBank Accession	*rbcL* Query Length	*rbcL* BOLD	*rbcL* GenBank	*matK* Sequencing Primer	*matK* GenBank Accession	*matK* Query Length	*matK* BOLD	*matK* GenBank	*trnH-psbA* Sequencing Primer	*trnH-psbA* GenBank Accession	*trnH-psbA* Query Length	*trnH-psbA* GenBank	*ITS2* Sequencing Primer	*ITS2* GenBank Accession	*ITS2* Query Length	*ITS2* GenBank
Angiosperms	Apiales	Apiaceae	*Conium*	*maculatum*	2005	4	rbcLaF/rbcLaR	MK895569	470	PP+	PP+	matK-KIM1R-F/matK-KIM3F-R	MK898780	456	PP	PP+	trnH2/psbAF	MK895611	252	PP	ITS2F	MK895637	309	PP+
	Aquifoliales	Aquifoliaceae	*Ilex*	*opaca*	NMNH	2	N/A	N/A	429	no match	PP	N/A	N/A	757	PP	PP	N/A	N/A	429	PP	N/A	N/A	N/A	N/A
	Arecales	Arecaceae	*Cocos*	*nucifera*	1951	2	rbcLaF/rbcLaR	MK895570	468	PP+	X	matK-KIM1R-F/matK-KIM3F-R	MK898781	490	PP+	PP+	psbAF	MK895607	336	PP	N/A	N/A	N/A	N/A
	Asparagales	Amaryllidaceae	*Amaryllis*	*belladonna*	1986	X	rbcLaF/rbcLaR	MK900738	456	PP	PP	N/A	N/A	N/A	N/A	N/A	N/A	N/A	N/A	N/A	N/A	N/A	N/A	N/A
	Asterales	Asteraceae	*Acamptopappus*	*shockleyi*	1976	4	rbcLaF/rbcLaR	MK895565	190	X	X	matK-KIM1R-F/matK-KIM3F-R	MK898777	483	X	X	trnH2/psbAF	MK895610	257	X	ITS2F/ITS4	MK895641	312	X
			Ambrosia	*artemisiifolia*	2003	X	rbcLaF/rbcLaR	MK900737	476	PP	PP+	N/A	N/A	N/A	N/A	N/A	N/A	N/A	N/A	N/A	N/A	N/A	N/A	N/A
			Artemisia	*cina*	1949	2	rbcLaF/rbcLaR	MK895573	485	P	P	matK-KIM1R-F/matK-KIM3F-R	MK898774	479	P	P	N/A	N/A	N/A	N/A	ITS2F/ITS4	MK895638	298	P
	Caryophyllales	Caryophyllaceae	*Agrostemma*	*githago*	1960	2	rbcLaF/rbcLaR	MK895562	465	PP	PP	matK390F/matK1326R	MK898775	393	PP	PP	trnH2/psbAF	MK895603	267	PP	N/A	N/A	N/A	N/A
	Cornales	Cornaceae	*Cornus*	*florida*	NMNH	2	N/A	N/A	553	PP	PP+	N/A	N/A	741	PP	PP	N/A	N/A	409	PP	N/A	N/A	N/A	N/A
		Hydrangeaceae	*Hydrangea*	*quercifolia*	2006	4	rbcLaF/rbcLaR	MK895581	465	PP	PP	matK-KIM1R-F/matK-KIM3F-R	MK898791	410	PP	PP	trnH2/psbAF	MK895619	339	PP	ITS2F/ITS4	MK895651	287	PP
	Fabales	Fabaceae	*Cercis*	*canadensis*	2006	4	rbcLaF/rbcLaR	MK895568	447	PP+	PP+	matK-KIM1R-F/matK-KIM3F-R	MK898779	476	X	X	trnH2/psbAF	MK895606	210	PP	ITS2F/ITS4	MK895644	313	PP+
			Erythrophleum	*suaveolens*	1987	4	rbcLaF/rbcLaR	MK895580	206	P	X	matK-KIM1R-F/matK-KIM3F-R	MK898790	440	PP+	PP+	trnH2/psbAF	MK895618	414	P	ITS2F/ITS4	MK895650	296	X
			Sophora	*tomentosa*	1977	4	N/A	N/A	432	PP+	PP+	N/A	N/A	338	PP	PP	N/A	N/A	573	PP+	ITS2F/ITS4	MK895666	310	PP
		Betulaceae	*Alnus*	*rubra*	1975	4	rbcLaF/rbcLaR	MK895564	464	PP+	PP+	matK390F/matK1326R	MK898783	494	PP+	PP+	trnH2/psbAF	MK895605	428	P	ITS2F/ITS4	MK895640	329	P
			Betula	*populifolia*	2006	2	rbcLaF/rbcLaR	MK895567	453	P	PP+	matK-KIM1R-F/matK-KIM3F-R	MK898778	452	PP+	PP+	N/A	N/A	N/A	N/A	ITS2F/ITS4	MK895643	316	P
		Fagaceae	*Quercus*	*michauxii*	2006	2	rbcLaF/rbcLaR	MK895592	467	P	PP+	matK-KIM1R-F/matK-KIM3F-R	MK898804	491	P	PP+	trnH2/psbAF	MK895615	508	P	N/A	N/A	N/A	N/A
				montana	1989	2	rbcLaF/rbcLaR	MK895593	477	X	X	matK-KIM1R-F/matK-KIM3F-R	MK898805	552	X	X	trnH2/psbAF	MK895631	305	X	N/A	N/A	N/A	N/A
				phellos	2006	2	rbcLaF/rbcLaR	MK895594	127	P	P	matK-KIM1R-F/matK-KIM3F-R	MK898806	472	P	P	trnH2/psbAF	MK895632	428	P	N/A	N/A	N/A	N/A
				rubra	NMNH	2	N/A	N/A	553	PP+	P	N/A	N/A	761	PP+	PP+	N/A	N/A	495	PP+	N/A	N/A	N/A	N/A
		Juglandaceae	*Carya*	*cordiformis*	NMNH	2	N/A	N/A	553	PP+	P	N/A	N/A	747	PP+	PP+	N/A	N/A	277	P	N/A	N/A	N/A	N/A
			Juglans	*nigra*	2006	4	rbcLaF/rbcLaR	MK895582	454	PP+	PP+	matK-KIM1R-F/matK-KIM3F-R	MK898792	458	PP	PP	trnH2/psbAF	MK895620	179	PP+	ITS2F/ITS4	MK895652	286	PP+
	Gentianales	Apocynaceae	*Cerbera*	*odollam*	2006	4	rbcLaF/rbcLaR	MK895571	467	X	X	matK-KIM1R-F/matK-KIM3F-R	MK898782	486	X	X	trnH2/psbAF	MK895608	342	X	ITS2F/ITS4	MK895645	322	X
			Nerium	*oleander*	1967	2	rbcLaF/rbcLaR	MK895586	476	PP+	PP	matK-KIM1R-F/matK-KIM3F-R	MK898796	506	PP+	PP+	trnH2/psbAF	MK895624	265	PP	N/A	N/A	N/A	N/A
			Thevetia	*peruviana*	1994	2	rbcLaF/rbcLaR	MK895602	478	PP+	PP+	matK-KIM1R-F/matK-KIM3F-R	MK898812	482	PP+	P	N/A	N/A	N/A	N/A	ITS2F/ITS4	MK895667	316	P
		Loganiaceae	*Strychnos*	*henningsii*	1989	4	rbcLaF/rbcLaR	MK895598	164	PP+	P	matK-KIM1R-F/matK-KIM3F-R	MK898809	493	P	P	trnH2/psbAF	MK895635	505	P	ITS2F/ITS4	MK895663	332	PP
				madagascariensis	2000	X	rbcLaF/rbcLaR	MK900739	453	PP+	PP+	N/A	N/A	N/A	N/A	N/A	trnHf05/psbA3F	MK900742	561	PP	ITS2F/ITS4	MK900746	292	P
				nux-vomica	N/A	4	rbcLaF/rbcLaR	MK895600	476	PP	PP	matK-KIM1R-F/matK-KIM3F-R	MK898810	546	PP	PP	trnH2/psbAF	MK895636	483	PP	ITS2F/ITS4	MK895664	330	PP
				spinosa	1994	4	rbcLaF/rbcLaR	MK895601	452	PP+	PP	matK-KIM1R-F/matK-KIM3F-R	MK898811	526	PP	PP	trnH2/psbAF	MK895617	519	PP	ITS2F/ITS4	MK895665	321	PP
				toxifera	NMNH	2	N/A	N/A	553	PP	PP	N/A	N/A	767	PP	PP	N/A	N/A	295	P	N/A	N/A	N/A	N/A
	Lamiales	Plantaginaceae	*Digitalis*	*grandiflora*	1951	4	rbcLaF/rbcLaR	MK895577	465	PP+	P	matK-KIM3F-R	MK898786	501	P	P	trnH2/psbAF	MK895612	353	PP	ITS2F/ITS4	MK895646	299	PP+
				lanata	1933	2	rbcLaF/rbcLaR	MK895578	478	PP	PP	matK-KIM1R-F/matK-KIM3F-R	MK898787	463	PP	PP	N/A	N/A	N/A	N/A	ITS2F/ITS4	MK895647	307	PP+
				purpurea	1959	4	rbcLaF/rbcLaR	MK895579	419	PP+	PP+	matK-KIM1R-F/matK-KIM3F-R	MK898788	449	PP	PP+	trnH2/psbAF	MK895613	349	P	ITS2F/ITS4	MK895648	304	PP+
	Laurales	Lauraceae	*Sassafras*	*albidum*	1999	4	rbcLaF/rbcLaR	MK895596	453	PP+	PP+	matK390F/matK1326R	MK898784	529	PP+	PP+	trnH2/psbAF	MK895633	367	PP	ITS2F/ITS4	MK895661	283	X
	Magnoliales	Magnoliaceae	*Liriodendron*	*tulipifera*	NMNH	2	N/A	N/A	553	PP	PP+	N/A	N/A	742	PP+	PP+	N/A	N/A	476	PP	N/A	N/A	N/A	N/A
			Magnolia	*virginiana*	2006	4	rbcLaF/rbcLaR	MK895585	479	PP+	PP+	matK-KIM1R-F/matK-KIM3F-R	MK898795	449	PP	PP	trnH2/psbAF	MK895623	373	PP+	ITS2F/ITS4	MK895654	297	X
	Malpighiales	Euphorbiaceae	*Ricinus*	*communis*	1973	4	rbcLaF	MK895595	494	X	X	matK-KIM1R-F/matK-KIM3F-R	MK898807	502	X	X	trnH2/psbAF	MK895616	439	X	ITS2F/ITS4	MK895660	324	X
		Salicaceae	*Populus*	*balsamifera*	NMNH	2	N/A	N/A	524	PP	PP	N/A	N/A	773	PP	PP	N/A	N/A	306	PP	N/A	N/A	N/A	N/A
				deltoides	2006	2	rbcLaF/rbcLaR	MK895588	465	PP+	PP+	matK390F/matK1326R	MK898799	423	PP+	P	N/A	N/A	N/A	N/A	ITS2F/ITS4	MK895657	282	PP+
	Malvales	Tilaceae	*Tilia*	*americana*	NMNH	2	N/A	N/A	525	PP+	PP+	N/A	N/A	764	PP+	PP+	N/A	N/A	N/A	N/A	N/A	N/A	N/A	N/A
	Myrtales	Lythraceae	*Lagerstroemia*	*indica*	1983	2	rbcLaF/rbcLaR	MK895583	465	PP+	PP+	matK-xf/matKMALPR1	MK898793	426	PP	PP+	trnH2/psbAF	MK895621	120	PP+	N/A	N/A	N/A	N/A
			Lythrum	*salicaria*	2006	4	rbcLaF/rbcLaR	MK895584	468	PP+	PP+	matK-KIM1R-F/matK-KIM3F-R	MK898794	493	PP+	PP+	trnH2/psbAF	MK895622	300	PP	ITS2F/ITS4	MK895653	316	PP+
	Poales	Poaceae	*Saccharum*	*brevibarbe*	1962	4	rbcLaF/rbcLaR	MK895574	447	P	P	matK390F/matK1326R	MK898789	428	P	P	trnH2/psbAF	MK895614	455	P	ITS2F/ITS4	MK895649	310	P
			Phragmites	*australis*	NMNH	2	N/A	N/A	553	PP	PP+	N/A	N/A	594	PP+	PP+	N/A	N/A	589	PP+	N/A	N/A	N/A	N/A
			Sorghastrum	*nutans*	1941	2	rbcLaF/rbcLaR	MK895599	414	X	PP+	matK454F/matK1315R	MK898785	532	PP	PP+	N/A	N/A	N/A	N/A	ITS4	MK895670	210	X
			Spartina	*cynosuroides*	1984	4	rbcLaF/rbcLaR	MK895597	480	P	P	matK390F/matK1326R	MK898808	417	P	P	trnH2/psbAF	MK895634	533	X	ITS2F/ITS4	MK895662	286	PP
	Proteales	Plantanaceae	*Platanus*	*occidentalis*	2006	4	rbcLaF/rbcLaR	MK895576	451	PP+	PP+	matK-KIM1R-F/matK-KIM3F-R	MK898801	477	PP+	PP+	psbAF	MK895628	294	PP	ITS2F/ITS4	MK895655	327	PP+
	Rosales	Rosaceae	*Prunus*	*serotina*	NMNH	2	N/A	N/A	524	PP	PP	N/A	N/A	670	PP	PP	N/A	N/A	360	PP	N/A	N/A	N/A	N/A
		Ulmaceae	*Ulmus*	*americana*	2006	4	N/A	N/A	553	PP	PP	N/A	N/A	781	PP	PP	N/A	N/A	281	PP	ITS2F/ITS4	MK895668	209	P
	Sapindales	Sapindaceae	*Acer*	*palmatum*	2006	4	rbcLaF/rbcLaR	MK895563	475	PP+	PP+	matK-KIM1R-F	MK898776	488	PP+	PP+	trnH2/psbAF	MK895604	391	PP	ITS2F/ITS4	MK895639	329	PP
	Sapindales			*rubrum*	NMNH	2	N/A	N/A	553	PP+	PP+	N/A	N/A	732	PP	PP	N/A	N/A	492	PP	N/A	N/A	N/A	N/A
	Solanales	Solanaceae	*Atropa*	*belladonna*	1981	4	rbcLaF/rbcLaR	MK895566	465	PP+	PP+	matK-KIM1R-F/matK-KIM3F-R	MK898773	408	PP	PP	trnH2	MK895609	127	PP	ITS2F/ITS4	MK895642	327	PP
Ferns	Dennstaedtiales	Dennstaedtiaceae	*Pteridium*	*aquilinum*	2007	4	rbcLaF/rbcLajf634R	MK895575	536	PP+	PP	polypodF1/polypodR1	MK898798	494	no match	PP	trnH2/psbAF	MK895626	410	PP	ITSS2F	MK895669	180	X
	Osmundales	Osmundaceae	*Osmunda*	*cinnamomea*	2001	X	N/A	N/A	N/A	N/A	N/A	N/A	N/A	N/A	N/A	N/A	trnh2	MK900743	344	PP	N/A	N/A	N/A	N/A
	Polypodiales	Dryopteridaceae	*Polystichum*	*acrostichoides*	2010	4	rbcLaF/rbcLaR	MK895587	461	PP+	PP	polypodR1	MK898797	482	X	P	trnH2/psbAF	MK895625	458	PP	ITS2F/ITS4	MK895656	264	X
Gymnosperm	Pinales	Cupressaceae	*Thuja*	*occidentalis*	NMNH	2	N/A	N/A	553	PP+	PP+	N/A	N/A	N/A	N/A	N/A	N/A	N/A	496	PP	N/A	N/A	N/A	N/A
		Pinaceae	*Pinus*	*strobus*	1991/2006	2	rbcLaF/rbcLajf634R	MK895590	519	PP+	PP+	Gym_F1A/Gym_R1A	MK898802	471	PP+	PP+	trnH2/psbAF	MK895629	557	PP	N/A	N/A	N/A	N/A
				taeda	2006	4	rbcLaF/rbcLajf634R	MK895591	505	PP+	PP+	Gym_F1A/Gym_R1A	MK898803	471	PP+	PP+	trnH2/psbAF	MK895630	535	PP+	ITS2F/ITS4	MK895659	326	P
				virginiana	1982	X	rbcLa-1/rbcLa-B	MK900740	104	PP+	PP+	N/A	N/A	N/A	N/A	N/A	N/A	N/A	N/A	N/A	ITS4R	MK900747	144	X
Mosses	Bryales	Bryaceae	*Pohlia*	*nutans*	1976	4	rbcLaF/rbcLaR	MK895589	444	X	X	moss404F/moss1336R	MK898800	265	X	X	trnH2/psbAF	MK895627	170	X	ITS2F/ITS4	MK895658	380	X
	Dicranales	Ditrichaceae	*Ceratodon*	*purpureus*	2001	2	rbcLaF/rbcLaR	MK895572	468	X	X	N/A	N/A	N/A	N/A	N/A	N/A	N/A	N/A	N/A	N/A	N/A	N/A	N/A
	Hypnales	Hylocomiaceae	*Pleurozium*	*schreberi*	1949	X	rbcLaF/rbcLaR	MK900741	416	PP+	PP	N/A	N/A	N/A	N/A	N/A	trnH2/psbAF	MK900744	147	PP	N/A	N/A	N/A	N/A

'X' denotes species not used in analysis, '2' denotes species included in 2-loci analysis (*rbcL* and *matK*), and '4' denotes species included in 4-loci analysis (*rbcL*, *matK*, *trnH-psbA* and *ITS2*). N/A=specimen did not amplify with any primer pair or had low quality sequence data, thus no downstream analyses were possible; X= misidentified at both the genus and species level; ✓= accurate genus level identification; ✓✓= unambiguous genus and species level identification; ✓✓+= ambiguous species level identification (*i.e.,* where records with the same top statistic match represent more than one species); N/A= sequences were provided by USNM (not uploaded into GenBank), thus year of collection, primer information, and all 4-loci sequences were not available.
